# Life Satisfaction Predicts Perceived Social Justice: The Lower Your Life Satisfaction, the Less Just You Perceive Society to Be

**DOI:** 10.3389/fpsyg.2020.540835

**Published:** 2020-11-24

**Authors:** Qifan Jia, Jie Zhou, Mingquan Huang

**Affiliations:** ^1^Key Laboratory of Behavioral Science, Institute of Psychology, Chinese Academy of Sciences, Beijing, China; ^2^Department of Psychology, University of Chinese Academy of Sciences, Beijing, China; ^3^School of Management, Guilin University of Aerospace Technology, Guilin, China

**Keywords:** life satisfaction, perceived social justice, happiness, distributive justice, procedure justice, uncertainty management model, random-intercept cross-lagged panel model, multiple group analysis

## Abstract

It has been well established that life satisfaction is related to perceived social justice. However, current theories provide contrary assumptions on the direction of the influence. In this research, we use data from two longitudinal surveys collected in China to test the reciprocal relations between life satisfaction and perceived social justice over time. With a random intercept cross-lagged panel model, we disaggregate the between-person effect and the within-person effect of the relationship. To specify the conditions of the effect, we consider income levels as the moderator. Study 1 (*N* = 119) showed that on the between-person level, life satisfaction, and perceived social justice are positively correlated. On the within-person level, cross-lagged effect results showed that an individuals’ deviations from their expected score in life satisfaction predict deviations from their expected perceived social justice at the next time point, while deviations from expected perceived social justice does not predict subsequent deviations from expected life satisfaction. In study 2 (*N* = 637), we divided participants into three groups based on their household income and conducted a multiple group analysis to test its moderation effect. We found that the between-person correlation of life satisfaction and perceived social justice is not moderated by income level, and it is significant in all the three groups. However, the within-person cross-lagged effect is moderated by income level, and the effect of life satisfaction on perceived social justice only exists in the low income group. This research confirms the unidirectional relationship between life satisfaction and perceived social justice across time, and clarifies the effect in different levels and income groups, providing new insights on the formation of justice perception. It is recommended that future studies apply experimental designs to reach causal effects and explore more possible moderators and mediators.

## Introduction

There is a great deal of evidence showing that inequality decreases happiness, subjective well-being, or life satisfaction (Schwarze and Härpfer, 2007; Wu and Li, 2013; Oishi and Kesebir, 2015). There is no doubt that there is a connection between objective social justice and life evaluation because the former cannot logically be changed by the latter, but does this relationship also apply to perceived social justice?

Life satisfaction is a cognitive judgmental process that assesses a person’s life qualities according to the criteria the person selects on their own (Diener et al., 1985). The standard an individual sets for him or herself is not externally imposed, however, it is much influenced by the social environment. Perceived social justice refers to people’s general appraisal of social justice. It contains both distributive justice, which is the fairness of allocation results (Deutsch, 1985), and procedural justice, which is concerned with the fairness of the allocation process (Thibaut and Walker, 1975). It has been suggested that justice judgments are the result of a rational-cognitive process whereby one person compares an outcome-to-input ratio to that of another (Adams, 1965), or by observing the process of distribution (Thibaut and Walker, 1975). Meanwhile, subjective-affective elements play an important role in forming justice judgment (Van Den Bos, 2003).

Previous studies on the relationship between life satisfaction and perceived social justice are insufficient due to three aspects. Firstly, current theories provide contrary assumptions on the direction of influence, and the existing correlation research is unable to provide a directional conclusion. Secondly, these studies indicate that people who have a high level of life satisfaction are more likely to have a high level of perceived social justice. However, this cross-sectional correlation does not inform us whether perceived social justice changes depending on life satisfaction change, or whether life satisfaction change influences perceived social justice change over time at the individual level. Thirdly, research in this field has ever considered the conditions of the reciprocal effect. Therefore, this study uses cross-lagged longitudinal studies to examine the relationship between perceived social justice and life satisfaction and explore the moderation effect of income level in this research.

### Within-Person Effect and Between-Person Effect

Cross-lagged panel models with longitudinal data provide a way to test the reciprocal relationship between life satisfaction and perceived social justice change over time. It allows for inferences of causality by comparing the relative effect of the two variables on each other across time (Wu et al., 2018), and reduces the probable parameter bias that cross-sectional data arises through the inclusion of the effect of the dependent variable at the previous time (Selig and Preacher, 2009).

This effect might only exist at the population level but not apply to a specific individual. The importance of disaggregating the between-person and the within-person level effect of the relationship is illustrated by Simpson’s Paradox (Kievit et al., 2013). Consider the following hypothetical example, where at the between-person level, a higher level of life satisfaction is associated with a higher level of perceived social justice, but the opposite or no relationship conclusions might be true for individual levels, meaning that an individual’s increase in life satisfaction is related to a decrease in perceived social justice or there is no correlation between them.

**FIGURE 1:**
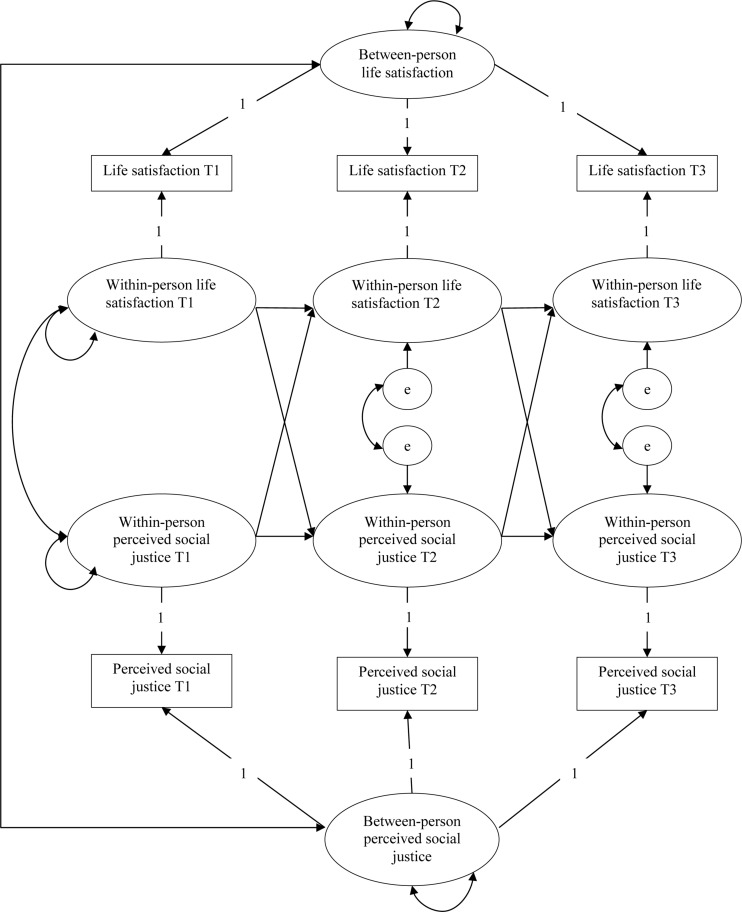
The random intercept cross-lagged panel model (RI-CLPM).

In our consideration of the individual differences among people, we decided to use a random intercept cross-lagged panel model (RI-CLPM), which decomposes each score into a between-person part and a within-person part and is regarded as an extension of the traditional cross-lagged panel model (Hamaker et al., 2015), see Figure 1 for the schematic representation. In this model, a time-invariant part is split out as the random intercept, which indicates the trait-like variability among people. The autoregressive paths reflect the extent of within-person deviations in life satisfaction and perceived social justice scores that can be explained by deviations from their expected life satisfaction and perceived social justice scores at previous time points, respectively. The concurrent correlation of the two variables at T1 reflects the correlation between deviations from their expected life satisfaction and perceived social justice. Associations at T2 and T3 reflect the correlation of the within-person change in life satisfaction and perceived social justice. Cross-lagged paths reflect the extent to which changes in an individual’s life or deviation from their expected life satisfaction scores is related to deviations from their expected perceived social justice scores at the previous time point, after controlling for the auto-regression effect and vice versa.

The between-person correlation between perceived social justice and life satisfaction can be explained from two perspectives. The “bottom-up” perspective outlines that an individual’s overall life satisfaction depends on his/her satisfaction with different life domains (Loewe et al., 2014), among which social justice is an important element of social life. Other research has found that people’s quality of life can be reflected by their concerns about social issues (Riggs and Turner, 2000). Perceived social justice as the perception of social conditions, is related to life satisfaction (Liao et al., 2005; Sun and Xiao, 2012). The “top-down” perspective claims that stable traits, like personality, predispose people to be satisfied with their lives and social conditions differently. Based on the results from Big Five, extraversion and neuroticism are strong predictors of life satisfaction at the global level (Schimmack et al., 2004). High extraversion and low neuroticism indicate high life satisfaction (Steel et al., 2008). Research on perceived social justice also provides evidence on the effect of personality, showing that neuroticism is negatively related to distributive justice perception (Bernerth et al., 2006). Therefore, we propose H1: that *people with a higher level of life satisfaction are more likely to report a higher level of perceived social justice than people with a lower level of life satisfaction.*

### A Reciprocal Relationship Between Perceived Social Justice and Life Satisfaction

Besides the correlation between life satisfaction and perceived social justice in the population, we are also interested in their reciprocal relationship within an individual. According to relative deprivation theory (Davis, 1959), people conduct social comparisons with their past, or with another person, group, their ideal, or some other social category. Individuals who make an unfavorable comparison and judge the outcome to be illegitimate and stable are said to be in a state of relative deprivation (Walker and Pettigrew, 1984). People form their life satisfaction perception by comparing their life with a standard. Perceived poor social justice implies a huge disparity among people and negative comparisons, especially for people who are in disadvantageous positions. Victims of negative social comparison evoke a strong sense of relative deprivation, leading to low life satisfaction. Therefore, we propose H2a: that *an increase in perceived social justice relative to a person’s average level of perceived social justice would predict an increase in their subsequent life satisfaction.*

While forming social justice perception, individuals in a society do not always have precise information about their actual social context (Oshio and Urakawa, 2014). According to the uncertainty management model, people tend to use other information as heuristic substitutes to assess fairness (Van Den Bos and Lind, 2002). For example, they may use justice perception about how they were/are treated as a substitute when they lack information about outcome justice (Van Den Bos et al., 1997). Furthermore, affective states are also an important substitute (Van Den Bos, 2003). In information-uncertain conditions, people who are high in negative affect are more likely to perceive a situation as hostile and unfair while people high in positive affect will see it as more favorable and fair (Barsky and Kaplan, 2007), and this effect is amplified by personal relevance (Mao et al., 2018). Although no direct evidence has ever addressed the effect of life satisfaction on perceived social justice, there is a reason to assume life satisfaction as an effective and efficient reference for justice judgment, because life satisfaction reflects the overall evaluation of one’s quality of life and it is the most relevant information people have in their life for reference. Therefore, we propose H2b: that *an increase in life satisfaction relative to a person’s average level of life satisfaction would predict an increase in their subsequent perceived social justice.*

### The Relationship Varies by Income Level

Life satisfaction in China has gone through a U-shaped swing over the last three decades (Easterlin et al., 2012, 2017; Clark et al., 2019), which has created a flowing perception of social justice among citizens. Inequality functions differently on people at distinct income levels. Oishi et al. (2011) found that for people in the lower 60% income group, their happiness is negatively related to income equality, and this relationship is mediated by perceived fairness. However, for people in the 60–80% income group, their happiness is higher in the years with more income inequality. And among the top-20% income group, perceived fairness is not associated with happiness.

Again, this effect does not disaggregate the between-person effect and the within-person effect. Given that the between-person correlation of life satisfaction and perceived social justice is more trait-like, it should be more significant among people with high incomes and stable living conditions. We predict that H3a: *on the between-person level, the relationship between life satisfaction and perceived social justice is moderated by income level, and the relationship is stronger in higher income groups.*

However, the within-person correlation is more state-like and more influenced by surrounding conditions. Since wealthy and rich people are the beneficiaries of social inequality, their fluctuation in life evaluation is relatively independent of social perception change. For example, even their perceptions of social perception deteriorate, they can still be satisfied with their life or even more satisfied. By contrast, their judgment of fairness might decrease even when their life satisfaction improves. However, this is not the case for poor people, who are at a disadvantage in terms of social inequality and whose lives are determined by the social environment. Therefore, their life perception and social perception are interrelated. We predict that H3b: *on the within-person level, the relationship between life satisfaction and perceived social justice is moderated by income level, and the relationship is stronger in lower income groups.*

These hypotheses were tested in two samples. Study 1 focused on primary and secondary teachers in China, who are facing great challenges including a high level of stress, inadequate breaks, and low salary (Liu and Onwuegbuzie, 2012), but whose perceptions of life and society are vital to the development, mental health, perception of the world, and values of the next generation (Braun et al., 2020). This sample provides an opportunity to test these hypotheses in a relatively narrow group. Study 2 extended the subjects to a community sample consisting of citizens of diverse genders, ages, and occupations, which allows for a more valid and robust conclusion.

## Study 1

### Methods

#### Participants

The first study used data from one of our previous programs named “Survey on teacher’s mental health in Puyang.” With snowball sampling, a total of 121 teachers from primary and middle schools participated in first wave data collection, and 119 remained in the second and the third wave (98.35% retention rate; 45 men and 74 women; age ranged 28–67, *M* = 42.05, *SD* = 8.32). Among them, 101 participants were married (84.9%), 10 were unmarried, 7 were divorced, and 1 was widowed. The majority reported that they had attended college (79, 66.4%), undertaken a bachelor’s degree (30, 25.2%), attended secondary school (8, 6.7%), and attended high school (2, 1.7%).

#### Measures and Procedure

Participants completed the same questionnaire at three time points in 2015, with a 1 month gap between each. Questionnaires were in Mandarin and were completed anonymously on paper. Informed consent was obtained from the participants before the first round of research. The questionnaires were 5 pages long, containing four parts: including sections on life evaluation, job evaluation, government evaluation, and demographic information. Life satisfaction and perceived social justice items belonged to the first part and were presented successively on the first page.

Life satisfaction was assessed with the five-item Satisfaction with Life Scale (SWLS; Diener et al., 1985). The answers were given on a four-point scale from 1 (strongly disagree) to 4 (strongly agree). The Cronbach’s α of this scale for the three data-collection points were 0.82, 0.77, and 0.69, respectively. The perceived social justice scale was adapted from Zhang and Zhou (2018) research and comprised two items. The first pertained to procedural justice (“Do you think the way social welfare and resources have been allocated in the last month is fair?”). The second was concerned with distributive justice (“Do you think the benefits and resources you have enjoyed over the last month are fair?”). Both questions used the same five-point scale, ranging from 1 (very unfair) to 5 (very fair). The Cronbach’s α of the perceived social justice scale for the three data-collection points were 0.94, 0.95, and 0.90, respectively. Demographic information included: gender, age, education level, marital status, and monthly household income (“What is the income level of your whole family in the last month, including salaries, income from part-time jobs, and any unfixed income, such as rental income, interest, and other material objects, such as wood, cotton, oil, meat, rice, etc.?”).

All the materials and procedures were approved by the institutional review board of the Institute of Psychology, Chinese Academy of Sciences.

### Results

#### Descriptive Statistics and Correlations

Descriptive statistics are presented in Table 1. The correlations of life satisfaction across three waves and the perception of social justice across three waves were significant (ranging from 0.21 to 0.52), which implied consistency and variability over time. There were significant positive correlations between perceived social justice and life satisfaction within and across waves.

**TABLE 1:** Means, standard deviations, and correlations in study 1.

	***M***	***SD***	**1**	**2**	**3**	**4**	**5**	**6**	**7**	**8**	**9**	**10**
1 Gender	1.62	0.49	–									
2 Age	42.05	8.32	0.28**	–								
3 Education	4.15	0.61	−0.03	0.24**	–							
4 Income	7.37	1.57	0.22*	–0.33**	0.15	–						
5 LS_1	1.64	0.48	−0.05	−0.22*	0.04	0.44**	–					
6 LS_2	1.58	0.43	0.04	−0.14	–0.01	0.41**	0.51**	–				
7 LS_3	1.58	0.36	−0.06	−0.17	–0.08	0.24**	0.34**	0.52**	–			
8 PSJ_1	1.74	0.89	−0.03	−0.14	0.15	0.31**	0.45**	0.35**	0.21*	–		
9 PSJ_2	1.54	0.79	−0.05	−0.21*	0.06	0.45**	0.41**	0.57**	0.24*	0.35**	–	
10 PSJ_3	1.63	0.78	0.10	−0.17	0.04	0.41**	0.37**	0.42**	0.24**	0.40**	0.45**	—

N = 119. *p < 0.05, **p < 0.01. LS 1–3 = Life satisfaction of time 1 to time 3. PSJ 1–3 = Perceived social justice of time 1 to time 3. Gender: 0 = male; 1 = female. Education: 1 = senior high school; 2 = secondary school; 3 = college; 4 = bachelor’s degree; and 5 = master’s degree. Monthly household income (current RMB): 1 = 1,000 or below; 2 = 1,001–2,000; 3 = 2,001–3,000; 4 = 3,001–4,000; 5 = 4,001–5,000; 6 = 5,001–6,000; 7 = 6,001–8,000; 8 = 8,001–10,000; 9 = 10,001–12,000; 10 = 12,001–15,000; 11 = 15,001–20,000; 12 = 20,001–30,000; 13 = 30,001–50,000; and 14 = 50,001 or above.

#### Confirmatory Factor Analysis

To determine and then confirm whether life satisfaction and perceived social justice were distinct from each other at the three time points, we undertook two factor analyses^1^. First, a single-factor model with all seven items loaded onto one factor at the three time points was conducted. Results showed a poor-fitting model, χ^2^(183) = 470.055, *p* < 0.001, CFI = 0.763, TLI = 0.728, RMSEA = 0.115, SRMR = 0.149. We then conducted a two-factor model, where life satisfaction and perceived social justice were loaded on two separate factors at the three time points. Results showed that the model had an excellent fit, χ^2^(171) = 215.729, *p* < 0.05, CFI = 0.963, TLI = 0.955, RMSEA = 0.047, SRMR = 0.058. The difference between the two models was significant, Δχ^2^(12) = 254.326, *p* < 0.001, which indicated that these two variables were distinct factors at all the time points.

#### Measurement Invariance Over Time

To verify that the effect across time is due to real changes in variables, it was necessary to assess the equivalence of these constructs across time. We followed the convention of doing measurement invariance with three steps (Putnick and Bornstein, 2016), see Table 2 for the results. The cutoff of a −0.01 change in CFI and 0.015 in RMSEA was set as the criterion (Chen, 2007). First, we established a configural invariance model based on the revised two-factor mode. This model is the baseline model without any constraints, and the result is described above, in the two-factor CFA test. Then we examined a weak invariance model, also known as the metric invariance model, with an item factor loading being equal over time. Results showed that the model fit [χ^2^(185) = 259.109, *p* < 0.001, CFI = 0.939, TLI = 0.931, RMSEA = 0.058, SRMR = 0.112] decreased significantly in comparison to the configured model, ΔCFI = −0.024, ΔRMSEA = 0.011. Therefore, we established a partial weak invariance by releasing the loading constraints of item 1 in the Satisfaction with Life Scale across the three times points. The model fit change was acceptable, ΔCFI = −0.007, ΔRMSEA = 0.003, supporting a partial weak invariance. Finally, we further tested the strong invariance, also known as the scalar invariance model, with intercepts being equal over time. The model fit change was acceptable, ΔCFI = −0.007, ΔRMSEA = 0.001, indicating a strong invariance over time.

**TABLE 2:** Measurement invariance over time in study 1.

**Model**	**χ^2^**	***df***	**CFI**	**RMSEA**	**TLI**	**SRMR**	**ΔCFI**	**ΔRMSEA**
Configural	215.729*	171	0.963	0.047	0.955	0.058		
Partial weak invariance^*a*^	236.852**	183	0.956	0.050	0.949	0.081	−0.007	0.003
Strong invariance	258.416**	197	0.949	0.051	0.946	0.076	−0.007	0.001

*N = 119. ^∗^*p* < 0.05, ^∗∗^*p* < 0.01. CFI, comparative fit index; RMSEA, Root mean square error of approximation; TLI, Tucker-Lewis fit index; SRMR, standardized root mean square residual. ^*a*^A model with factor loading of all the items set equal over time, except for item 1 in the Satisfaction with Life Scale.*

#### Random Intercept Cross-Lagged Panel Model

Given sufficient measurement invariance over time, we investigated the reciprocal relationship between life satisfaction and perceived social justice. The intra-class coefficient (ICC) for life satisfaction was 0.71, indicating that 71% of the variance was due to between-person differences and 29% was because of the fluctuations within persons. Thirty-four percent of the variance in perceived social justice was explained by intra-individual differences (ICC = 0.66).

**FIGURE 2:**
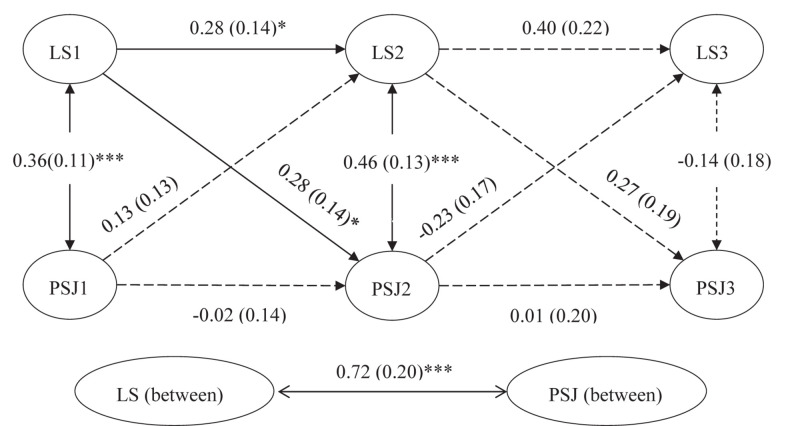
Simplified random intercept cross-lagged panel model (RI-CLPM) in study 1. ^∗^*p* < 0.05, ^∗∗∗^*p* < 0.001. *N = 119*. LS 1–3 = life satisfaction of time 1 to time 3. PSJ 1–3 = perceived social justice of time 1 to time 3. Completely standardized parameter estimates are reported, with standard errors presented in parentheses.

We then conducted a random intercept cross-lagged panel model (RI-CLPM) with Mplus 7.4. Results in Figure 2 showed that the RI-CLPM had an acceptable fit, χ^2^(1) = 2.830, *p* = 0.093, RMSEA = 0.124, CFI = 0.990, TLI = 0.852, SRMR = 0.031. The between-person association between life satisfaction and perceived social justice was strong and positive (β = 0.72, *p* < 0.001), indicating that individuals with higher life satisfaction across the measurement waves reported higher perceived social justice than individuals with lower life satisfaction, which was consistent with H1.

On the within-person level, a significant concurrent association was found between life satisfaction and perceived social justice (β = 0.36, *p* < 0.001), meaning that individuals who scored higher than their expected life satisfaction score tended to score higher than their expected perceived social justice at T1. At T2, individuals whose life satisfaction changed also tended to change in perceived social justice (β = 0.46, *p* < 0.001). However, this was not the case for T3. The autoregressive paths indicated that within-person deviations in life satisfaction at T2 were predicted by deviations from the expected scores for life satisfaction at T1 (β = 0.28, *p* < 0.05). Other autoregressive paths were not significant.

In terms of within-person cross-lagged effect, we found that life satisfaction T1 significantly predicted perceived social justice T2 (β = 0.28, *p* < 0.05), indicating that individuals that deviated from their expected perceived social justice at T2 were predicted by deviations from their expected score in life satisfaction at T1. However, deviations of perceived social justice did not predict subsequent deviations of life satisfaction (β = 0.13, *p* = 0.31 for T1 to T2; β = −0.23, *p* = 0.18 for T2 to T3). H2b was partially supported.

### Discussion

Above all, we found that life satisfaction and perceived social justice were positively correlated on the between-person level. On the within-person level, life satisfaction predicted subsequent perceived social justice, but not vice versa. H1 and H2b were confirmed. However, there are several concerns in this study. First, the sample size is small, which makes the effect less robust and gives us no chance to separate different income groups to test the moderation effect. Second, we found that life satisfaction and perceived social justice in all three waves were significantly lower than the middle value (for life satisfaction, *t*s = −19.40, −23.32, −28.21, *p*s < 0.01; for perceived social justice, *t*s = −15.37, −20.11, −19.23, *p*s < 0.01), which might be a potential antecedent of the effect in H2b. Third, perceived social justice was assessed just after life satisfaction was assessed, and the close position of these two questionnaires might be the trigger of the effect. Therefore, we wish to examine the hypotheses with a larger sample within the consideration of these questions and test the moderation effect of income level in study 2.

## Study 2

### Methods

#### Participants

Study 2 used data from another of our previous programs named “Survey on people’s social attitude in Dongguan.” We recruited 978 citizens from Dongguan in Guangzhou province. At time 1, all the participants finished the survey (100% retention rate). At time 2, 800 participants finished the survey (81.80% retention rate). At time 3, 706 participants finished the survey (72.19% retention rate). At time 4, 637 participants finished the survey (65.13% retention rate). We compared the valid data (*N* = 637) with the missing data (*N* = 341) in terms of demographics and constructs of interest. The results indicated no significant difference between the two samples on life satisfaction, perceived social justice, age, gender, education, hukou, ethnicity, occupation, political status, and religion. However, people who completed the whole data collection process had a much higher household income than people who failed to finish the research (*t* = 21.87, *p* < 0.01). See Supplementary Appendix for detailed results of the different tests.

The final sample consisted of 312 men and 325 women. Age ranged from 18 to 70, *M* = 37.02, *SD* = 12.33. Han Chinese accounted for 95.4%. The rural population was 68.3%. The distribution of education level was: illiterate (16, 2.5%), primary school (88, 13.8%), junior high school (207, 32.5%), senior high school (179, 28.1%), secondary school (75, 11.8%), college (42, 6.6%), and bachelor’s degree (30, 4.7%). A wide range of occupations was covered, including institutional staff, government officials, enterprise workers, self-employed, farmers, migrant workers, students, freelancers, retirees, and the unemployed.

#### Measures and Procedure

Participants completed the same questionnaire at four time points in 2011, with a 1 month gap between each. The questionnaires were 14 pages long, containing four parts: life evaluation, community evaluation, social and government evaluation, and demographic information. Life satisfaction belonged to the first part and the items were presented on the first page. Perceived social justice belonged to the third party and the items were presented on the 10th page.

Items of life satisfaction and perceived social justice were the same as study 1, except that life satisfaction was assessed on a seven-point Likert scale from 1 (strongly disagree) to 7 (strongly agree). The Cronbach’s α of life satisfaction scale for the four data collection points were 0.82, 0.87, 0.89, and 0.87, respectively. The Cronbach’s α of the perceived social justice scale was 0.64, 0.67, 0.68, and 0.79.

All the materials and procedures were approved by the Institutional review board of the Institute of Psychology, Chinese Academy of Sciences.

### Results

#### Descriptive Statistics and Correlations

Descriptive statistics are presented in Table 3. The correlations of life satisfaction and perceived social justice across four waves were significant, ranging from 0.18 to 0.43. The relationship between perceived social justice and life satisfaction within and across waves was positive.

**TABLE 3:** Means, standard deviations, and correlations in study 2.

	***M***	***SD***	**1**	**2**	**3**	**4**	**5**	**6**	**7**	**8**	**9**	**10**	**11**	**12**
1 Gender	0.51	0.50	–											
2 Age	37.02	12.33	0.00	–										
3 Education	3.71	1.35	–0.11**	–0.07	–									
4 Income	3,713	1,913	0.02	0.01	0.34**	–								
5 LS_1	3.78	1.10	0.03	0.13**	0.08*	0.03	–							
6 LS_2	3.95	1.22	0.07	0.05	–0.03	0.10*	0.27**	–						
7 LS_3	4.08	1.13	0.06	0.08*	–0.04	–0.04	0.23**	0.43**	–					
8 LS_4	4.05	1.08	0.09*	0.10*	0.03	0.22**	0.20**	0.37**	0.33**	–				
9 PSJ_1	2.83	0.67	–0.00	0.12**	0.14**	0.03	0.21**	0.15**	0.12**	0.11**	–			
10 PSJ_2	2.90	0.68	0.04	0.02	0.09*	0.16**	0.17**	0.36**	0.23**	0.22**	0.23**	–		
11 PSJ_3	2.72	0.75	0.05	0.13**	0.07	–0.00	0.03	0.18**	0.29**	0.13**	0.20**	0.29**	–	
12 PSJ_4	2.78	0.75	–0.01	0.11**	0.06	–0.02	0.13**	0.19**	0.17**	0.20**	0.18**	0.32**	0.33**	–

N = 637. *p < 0.05, **p < 0.01. LS 1–4 = life satisfaction of time 1 to time 4. PSJ 1–4 = perceived social justice of time 1 to time 4. Gender: 0 = male; 1 = female. Education: 1 = illiteracy; 2 = primary school; 3 = junior high school; 4 = senior high school; 5 = secondary school; 6 = college; 7 = bachelor’s degree; 8 = master’s degree; and 9 = doctor’s degree.

#### Confirmatory Factor Analysis

Two confirmatory factor analyses^2^ were conducted to identify the structure of life satisfaction and perceived social justice at the four time points. The single-factor model indicated a poor fit, χ^2^(340) = 1,773.540, *p* < 0.001, CFI = 0.827, TLI = 0.807, RMSEA = 0.081, SRMR = 0.079, whereas the two-factor model reported an excellent fit, χ^2^(318) = 800.006, *p* < 0.001, CFI = 0.942, TLI = 0.931, RMSEA = 0.049, SRMR = 0.042. The difference between the two models was significant, Δχ^2^(22) = 973.534, *p* < 0.001, indicating that these two variables were distinct factors at all the time points.

#### Measurement Invariance Over Time

The same procedures were conducted to test measurement invariance as study 1, see Table 4 for the results. The configural invariance model had a good fit as described above in the two-factor CFA test. The weak invariance model with all factor loadings being equal over time also reported a good fit and the change of this model was not significantly different from the configural model, ΔCFI = −0.006, ΔRMSEA = 0.000. However, the model fit of strong invariance [χ^2^(360) = 1129.943, *p* < 0.001, CFI = 0.907, TLI = 0.902, RMSEA = 0.058, SRMR = 0.066] decreased significantly, ΔCFI = −0.029, ΔRMSEA = 0.009. Therefore, we established a partial strong invariance by releasing the loading constraints of items 3, 4, and 5 in the Satisfaction with Life Scale across the four times. The model fit change was acceptable, ΔCFI = −0.007, ΔRMSEA = 0.002, supporting a partial strong invariance.

**TABLE 4:** Measurement invariance over time in study 2.

**Model**	**χ^2^**	***df***	**CFI**	**RMSEA**	**TLI**	**SRMR**	**ΔCFI**	**ΔRMSEA**
Configural	800.006***	318	0.942	0.049	0.931	0.042		
Weak invariance	866.647***	339	0.936	0.049	0.929	0.057	−0.006	0.000
Partial strong invariance^*a*^	937.093***	351	0.929	0.051	0.924	0.060	−0.007	0.002

***p < 0.001. N = 637. RMSEA, Root mean square error of approximation; CFI, comparative fit index; TLI, Tucker-Lewis fit index; SRMR, standardized root mean square residual. ^*a*^A model with intercepts of all the items set equal over time, except for item 3, 4, and 5 in the Satisfaction with Life Scale.

#### The Random Intercept Cross-Lagged Panel Model and Multiple Group Analysis

**FIGURE 3:**
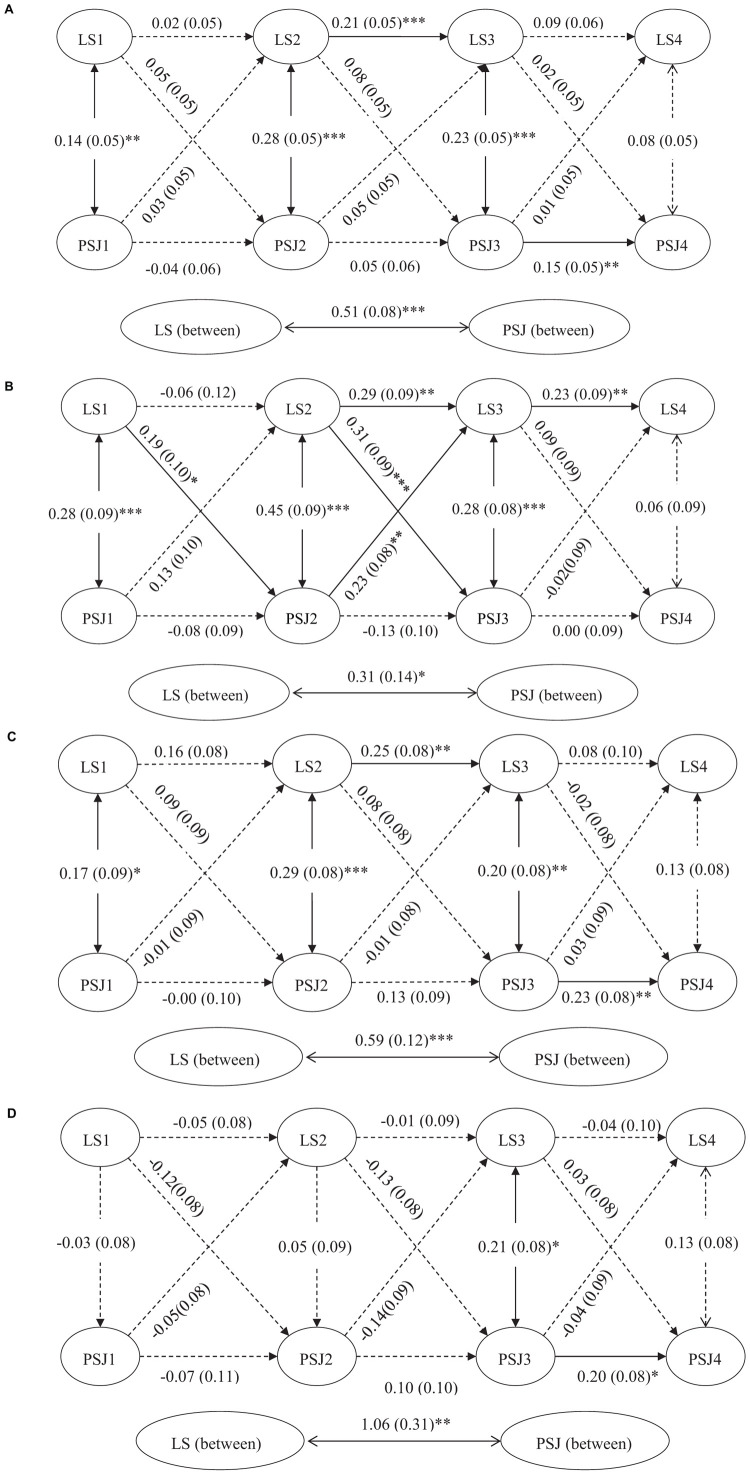
Simplified random intercept cross-lagged panel model (RI-CLPM) in study 2. *p < 0.05, **p < 0.01, ^∗∗∗^*p* < 0.001. **(A)** The whole sample, *N* = 637. **(B)** Low-income group, *N* = 198. **(C)** Middle-income group, *N* = 226. **(D)** High-income group, *N* = 213. LS 1–3 = life satisfaction of time 1 to time 3. PSJ 1–3 = perceived social justice of time 1 to time 3. Completely standardized parameter estimates are reported, with standard errors presented in parentheses.

The ICCs for life satisfaction and perceived social justice revealed that 37% of the variance of life satisfaction was due to within-person differences (ICC = 0.63) and 42% of the variance of perceived social justice was explained by intra-individual differences (ICC = 0.58). Similarly, to investigate the relationship between life satisfaction and perceived social justice, we adopted the same RI-CLPM as study 1. The results seen in Figure 3A show that the RI-CLPM for the whole sample had an acceptable fit, χ^2^ (9) = 33.325, *p* < 0.001, RMSEA = 0.065, CFI = 0.964, TLI = 0.889, SRMR = 0.044. The between-person association between life satisfaction and perceived social justice was significantly positive (β = 0.51, *p* < 0.001), indicating that individuals with higher life satisfaction had higher perceived social justice. However, on the within-person level, neither of the deviations from expected life satisfaction score predicted deviations of perceived social justice scores at the subsequent time points, nor did the deviations from the expected perceived social justice scores predict subsequent deviations in life satisfaction scores.

To examine the moderation effect of income level, we performed a multiple group analysis. Participants were divided into 3 groups according to their average household income. A total of 198 participants (31.08%, income range 637.5–2,475) were placed in the low-income group, 226 participants in the middle-income group (35.48%, income range 2,500–4,225), and 213 participants in the high-income group (33.44%, income range 4234.38–13,625).

Firs, we conducted an ICC test and RI-CLPM model for the three income groups separately. Results showed that the variance of life satisfaction and perceived social justice in the three groups were explained by the between and the within differences (in the low-income group, ICC = 0.70 for life satisfaction, ICC = 0.52 for perceived social justice; in the middle-income group, ICC = 0.68 for life satisfaction, ICC = 0.66 for perceived social justice; in the high-income group, ICC = 0.24 for life satisfaction, ICC = 0.53 for perceived social justice). All the three RI-CLPM models had an acceptable fit [in the low-income group, χ^2^(9) = 10.654, *p* = 0.300, RMSEA = 0.030, CFI = 0.993, TLI = 0.980, SRMR = 0.037, see Figure 3B; in the middle-income group, χ^2^(9) = 10.069, *p* = 0.345, RMSEA = 0.023, CFI = 0.996, TLI = 0.989, SRMR = 0.042, see Figure 3C; in the high-income group, χ^2^(9) = 17.002, *p* < 0.05, RMSEA = 0.065, CFI = 0.935, TLI = 0.799, SRMR = 0.046, see Figure 3D].

We then established a baseline model and three constrained models to examine the moderation effect of income level (see Table 5). The baseline model (unconstrained model; M1) included the three sub-samples at the same time with all the structural paths freely estimated for each group, and this model showed a good fit. In the M2 (Partially constrained B), we constrained the between-person correlation path between life satisfaction and perceived social justice among three income groups to be equal. The results showed that this model had a good fit and that there was no significant difference between M1 and M2, Δχ^2^(2) = 2.312, *p* = 0.315. H3a was rejected. In the M3 (Partially constrained W), we constrained the within-person cross-lagged correlation paths between life satisfaction and perceived social justice among three income groups. Results showed that this model had an acceptable fit, but there was a significant drop in the fit compared to M1, Δχ^2^(24) = 54.080, *p* < 0.001. We also established the M4 (fully constrained model) with all the paths constrained to equal among three groups, and the model fit was significant from M1, Δχ^2^(26) = 59.946, *p* < 0.001.

**TABLE 5:** Multiple group analysis in study 2.

	**χ*^2^***	***df***	**CFI**	**TLI**	**RMSEA**	**SRMR**	**Δχ*^2^***	**Δ*df***
M1 (three groups)	37.725	27	0.984	0.951	0.043	0.042		
M2 (three groups)	40.037	29	0.984	0.953	0.042	0.046	2.312^*a*^	2
M3 (three groups)	91.805***	51	0.940	0.901	0.061	0.069	54.080***^b^	24
M4 (three groups)	97.671***	53	0.934	0.896	0.063	0.080	59.946***^c^	26
M1 (low vs. middle)	20.723	18	0.995	0.985	0.027	0.039		
M3 (low vs. middle)	43.818*	30	0.975	0.954	0.047	0.057	23.095*^d^	12
M1 (low vs. high)	27.656	18	0.974	0.920	0.051	0.042		
M3 (low vs. high)	73.057***	30	0.885	0.786	0.084	0.077	45.401***^e^	12
M1 (middle vs. high)	27.071	18	0.979	0.934	0.048	0.044		
M3 (middle vs. high)	40.439	30	0.976	0.955	0.040	0.056	13.368^*f*^	12

*p < 0.05, ***p < 0.001. N _*to*__*tal*_ = 637, N _*low*_ = 198, N _*middle*_ = 226, N _*high*_ = 213. M1 refers to an unconstrained model; M2 refers to a partially constrained model in which between-person correlation is set to be equal; M3 refers to a partially constrained model in which within-person cross-lagged correlations are set to be equal; M4 refers to a fully constrained model. ^*a*–*c*^M2-M4 (three groups) compared with M1 (three groups), respectively. ^*d*–*f*^M3 compared with M1 among the same two income groups.

Finally, given the significant difference between M3 and M1, we used pairwise comparisons to determine which income group differences were significant on the within-person cross-lagged correlations. We conducted three unconstrained models with all the structural paths freely estimated and three partially constrained models with the within-person cross-lagged paths set to be equal among the two compared groups (low vs. middle; low vs. high; middle vs. high) (see Table 5). Results showed that while there was constraining for the low and the middle income groups [Δχ^2^(12) = 23.095, *p* < 0.05] or the low and the high income groups [Δχ^2^(12) = 45.401, *p* < 0.001], there was a significant drop in the fit. However, there was no significant drop in the fit when we constrained the middle and the high income groups [Δχ^2^(12) = 13.368, *p* = 0.34]. From Figure 3, we found that in the low income group, deviations from their expected life satisfaction scores predicted deviations from their expected perceived social justice scores at the subsequent time point (β = 0.19, *p* < 0.05 for T1–T2; and β = 0.31, *p* < 0.001 for T2–T3), while deviations from their expected perceived social justice scores only predicted subsequent deviations of life satisfaction scores from T2 to T3 (β = 0.23, *p* < 0.01), H2b was confirmed. All the cross-lagged paths were not significant in the middle and the high income groups. Above all, H3b was supported.

### Discussion

We found that income level was moderated the relationship between life satisfaction and perceived social justice on the within-person level but not on the between-person level. Deviations of life satisfaction predicted subsequent deviations of perceived social justice in the low income group, but it did not apply to the middle and the high income groups.

These results extend the findings of a study by Oishi et al. (2011) on the effect of income. After integrating between-person and within-person effects among different income groups in this research, we found that the correlation between life satisfaction and perceived social justice is a trait-like relationship for people with middle and high income. This might be because people with high or middle level incomes have more resources in society and they are better able to adapt their life and social conditions. Therefore, their life satisfaction and perceived social justice, as evaluations of these conditions, respectively, are more stable, and correlate with each other in a more robust way among the population. However, people with low incomes are the victim of inequality and their living conditions are closely bound to social conditions. Therefore, perceptions of their life and society are time-variant variables and are correlated with each other in a fluctuating way.

In terms of the reciprocal relationship between life satisfaction and perceived social justice across time, the conclusion we drew is contrary to the commonly accepted position adopted by most literature (Liao et al., 2005; Oishi et al., 2011; Sun and Xiao, 2012), that perception of social context determines life evaluation. We argue that this assumption is possibly based on the effect of objective social contexts on life evaluation and that perceived social contexts might not function in that process. First, perceptions can be obscure and inaccurate, being far away from the true reality. Using data from 60 counties in China, Zhou and Xie (2016) found no significant effect of regional differences in economic development on life satisfaction, as most people do not experience regional inequality personally: even those who are knowledgeable about regional inequality are not sensitive to it (Wang, 2008). Second, life satisfaction can be influenced by objective social context through a more unconscious path, with little conscious attention or monitoring (Stapel and Blanton, 2004), which such as rising crime rate (Coccia, 2018) and decreasing social trust (Delhey and Dragolov, 2014) caused by high inequality. This automatic process can be conducted without the judgment of social conditions.

When people do judge social justice, life satisfaction functions as a substitute to assess fairness according to our results. In this study, we found that people with low incomes are more likely to adopt life satisfaction to evaluate society. This might be because the life of low-income people is much more influenced by social justice, leading to a higher relevance in comparison with high-income people. This effect might function through a dual-process (Fiske and Taylor, 1991), combining a controlled and deliberate cognitive process, with which people refer to life satisfaction as social context, and an automatic and implicit process, where negative emotional responses, like grievances, were associated with low life satisfaction in the low-income group.

## General Discussion

In this research, we examined the relationship between life satisfaction and perceived social justice. The results of Study 1 confirmed that the positive relationship on the between-person level and the unidirectional relationship on the within-person level, which is that life satisfaction predicts subsequent perceived social justice but not vice versa. Study 2 tested the effects of income levels, showing that income level moderates the within-person effect but not the between-person effect. Correlation on the between-person level is significant in all the three income groups, but cross-lagged correlations on the within-person level are only significant in the low income group. Life satisfaction predicts subsequent perceived social justice but not vice versa, and this effect only exists in people with low incomes.

An exploration of the fact that within-person cross-lagged correlation exists in the whole sample of study 1, but not study 2, could be that life satisfaction, perceived social justice, and income level for participants in study 1 were all at a lower level. This is consistent with previous research showing that primary and secondary teachers are suffering great challenges from work. They work more hours, under greater pressure, but obtain less respect than before (Li et al., 2018). This should attract more attention and needs to be addressed because the happiness of teachers is vital for the healthy development of students and the progress of society. The improvement of teachers’ welfare and the construction of a complete mental healthcare system in school are imperative.

This research is inspiring in three aspects. Firstly, we found the unidirectional relationship between life satisfaction and perceived social justice within individuals. Although it is not a new idea that justice perception is a hybrid of justice information and other substitute information, few studies have considered the effect of life satisfaction on perceived social justice. Secondly, by adopting RI-CLPM, we can disaggregate the between-person and within-person effect, which contributes to our understanding of the relationship between these two variables. Thirdly, the incorporation of income levels enriches the research and specifies the within and between person effect among different conditions.

Our findings are beneficial in practice. An abundance of research showed that perception of social justice, as a source of political trust (Levi and Stoker, 2000), provides legitimacy of social institutions (Wegener, 2000), and influences the public’s cooperation with government (Zhang, 2017). China is currently actively addressing gaps in wealth, urban-rural differences, and imbalances in regional development. Based on our findings, the improvement of objective social justice is no longer the only way to improve perceived social justice. Caring for people’s lives and improving their life satisfaction, especially people with low incomes is also vital for the issue.

This research is not without limitations. The cross-lagged longitudinal design enables us to get closer to identifying causal relation rather than cross-sectional correlation, but it is still far from drawing a causal conclusion. Future studies should apply experimental designs to reach causal effects. Another direction for future studies could be the exploration of mediators and moderators of the effect of life satisfaction on perceived social justice. In this research, we found that income level moderates the effect and proposed that emotional response might be the mechanism for the cross-lagged effect in the low-income group. Whether life satisfaction functions directly by being a reference itself or indirectly by evoking affective factors, and whether there are factors other than income level that moderate these effects, still need to be further explored in future studies.

## Data Availability Statement

The datasets and analysis code for this study can be found in Open Science Framework (https://osf.io/h4nx2/).

## Ethics Statement

The studies involving human participants were reviewed and approved by the Ethics Committee for Scientific Research, Institute of Psychology, Chinese Academy of Sciences. The patients/participants provided their written informed consent to participate in this study.

## Author Contributions

QJ and JZ contributed to the conception and design of the study. MH organized the database. QJ performed the statistical analysis and wrote the first draft of the manuscript. All authors contributed to manuscript revision, read and approved the submitted version.

## Conflict of Interest

The authors declare that the research was conducted in the absence of any commercial or financial relationships that could be construed as a potential conflict of interest.

## Footnotes

1 In consistent with Bai et al. (2011)’s research, we found that item 4 and 5 in the Satisfaction with Life Scale shared a distinct meaning with the wording effect of the meaning of “past life.” Allowing residuals of these two items correlated improved the model fit significantly. Therefore, we adopted the same approach used in that research to revise the model with residuals of item 4 and 5 being correlated in the two models.

2 Consistent with study 1, we allowed residuals of item 4 and 5 in the Satisfaction with Life Scale correlated in the two models.
