# Supplementary material for: Life Satisfaction Predicts Perceived Social Justice: The Lower Your Life Satisfaction, the Less Just You Perceive Society to Be
Source: Front Psychol. 2020 Nov 24;11:540835. doi: 10.3389/fpsyg.2020.540835 (PMC7732682; doi:10.3389/fpsyg.2020.540835)
Supplement: Supplementary file 1 [file Data_Sheet_1.pdf]

Appendix. Difference tests between valid and invalid data

In study 1, a total of 121 people participated in the first wave of data collection, and 119 participated in the second and the third wave. The retention rate was 98.35%, with no need to test the difference between valid data and missing data.

In study 2, a total of 978 people participated in the first wave of data collection, and 637 remained in the fourth wave. Table 6 below showed the detailed retention rate at each time point. Table 7 and Table 8 represented the difference tests between valid data and missing data. We found that there was no significant difference between valid and invalid data on demographics and constructs of interest, except for average household income. People completed the whole data collection process had much higher household income than people who failed to finish the research.

Table 6 Retention rate in study 2

	Finished sample	Missed sample	Retention rate
Time 1	978		100%
Time 2	800	178	81.80%
Time 3	706	94	72.19 %
Time 4	637	69	65.13 %

Table 7 T test of continuous variables in study 2

	Valid		Invalid		t	df	p
	M	SD	M	SD			
LS_1	3.78	1.10	3.72	1.02	0.78	976	0.44
LS_2	3.95	1.22	3.93	1.11	0.58	798	0.56
LS_3	4.08	1.13	3.92	1.00	1.14	704	0.25
LS_4	4.05	1.08	—	—	—	—	—
PSJ_1	2.83	0.67	2.84	0.66	-0.24	976	0.81
PSJ_2	2.90	0.68	2.82	0.77	1.25	798	0.21
PSJ_3	2.72	0.75	2.86	0.69	-1.41	704	0.16
PSJ_4	2.78	0.75	—	—	—	—	—
Age	37.02	12.33	37.94	12.99	-1.09	976	0.27
Income	3713	1913	1316	1084	21.87**	994	0.00

Note. ** $p < 0.01$. N_{total} =978, N_{valid} = 637, $N_{invalid}$ = 341. LS 1-4 = life satisfaction of time 1 to time 4. PSJ 1-4 = perceived social justice of time 1 to time 4.

Table 8 Chi-square test of discrete variables in study 2

Variables	Items	Samples			$\chi2$	*df*	*p*
		Valid	Invalid	Total			
Gender	Male	312	169	481	0.03	1	0.86
	Female	325	172	497			
Education	Illiterate	16	5	21	8.00	6	0.24
	Primary school	88	57	145			
	Junior high school	207	109	316			
	Senior high school	179	84	263			
	Secondary school	75	43	118			
	College	42	33	75			
	Bachelor's degree	30	10	40			
Hukou	Urban	202	100	302	0.59	1	0.44
	Rural	435	241	676			
Ethnic	Han	608	319	927	1.62	1	0.20
	Others	29	22	51			
Occupation	Farmer	88	52	140	5.36	9	0.80
	Enterprise worker	89	51	140			
	Institution staff	60	36	96			
	Government official	7	7	14			
	Self-employed	140	70	210			

	Migrant worker	101	50	151			
	Retiree	16	13	29			
	Unemployed	34	16	50			
	Freelancer	87	41	128			
	Student	15	5	20			
Political status	No party identification	548	299	847	1.47	3	0.69
	League member	32	18	50			
	Communist Party of China member	56	24	80			
	Democrat	1	0	1			
Religion	No religious identification	286	136	422	3.50	4	0.48
	Buddhism	334	193	527			
	Taoism	3	3	6			
	Christianity	13	9	22			
	Islam	1	0	1			
Total		637	341	978			

Note. N_{total} =978, N_{valid} = 637, $N_{invalid}$ = 341.
